# Neurosurgery abroad? Medical Graduate's perspective from LMIC

**DOI:** 10.1016/j.bas.2023.102710

**Published:** 2023-11-08

**Authors:** Minaam Farooq, Sunaina Tariq, Shah Gul Zahra, Oday Atallah, Bipin Chaurasia

**Affiliations:** Department of Neurological Surgery, Weill Cornell Brain and Spine Center, Weill Cornell Medicine, New York-Presbyterian Hospital, New York, NY, USA; King Edward Medical University, Lahore, Pakistan; Hannover Medical School, Hannover, Germany; Department of Neurosurgery, Neurosurgery Clinic, Birgunj, Nepal

Neurosurgery is a specialized medical field that focuses on the surgical treatment of conditions related to the nervous system, which includes the brain, spinal cord, and peripheral nerves. Neurosurgeons work closely with other medical professionals, including neurologists, radiologists, and anesthesiologists, to provide comprehensive care to patients with neurological disorders. The decision of choosing a medical specialty after finishing the MBBS is undoubtedly a pivotal moment in a doctor's life. In the overall ranking of motives for specialty choice, family (71%), leisure time (66%) and job opportunities (48%) are rated more important than income (36%), mentoring (20%), status or scientific work (20%) (Abendroth et al., 2014).

When discussing neurosurgery, it's important to note that it falls within the realm of surgical subspecialties. So, doctors having fascination for surgery might opt it. It is a very prestigious specialty. The interest in the specialty is motivated by its popularity and remuneration. (Kanmounye et al., 2022). In today's era of modern medicine, neurosurgery stands as a challenging and rewarding field, fostering opportunities for interdisciplinary collaboration. The field has vast potentials of technological advancements, as discussed later. Furthermore, aside from other considerations, neurosurgery can be an appealing choice for physicians seeking a high degree of patient interaction, in contrast to specialties like anesthesia and radiology. Additionally, the prospect of a competitive salary is another compelling reason for selecting neurosurgery as a residency.

As one respondent of a study put it: ‘Neurosurgery is more than surgery; it is a complete art form. It requires discipline, attention to detail, and dexterity, but it also requires creativity. The neurosurgeon uses the scalpel just like the painter uses his brush, delicately, methodically, and with great attention to detail.’ (Kanmounye et al., 2022).

Numerous graduates choose to pursue neurosurgery abroad due to a multitude of captivating reasons. According to a research paper, the main reason for wanting to train abroad was to get better training (68.5%). Among the common intraoperative neurosurgical tools used in Pakistan, the microscope and endoscope are the most frequently used instruments by neurosurgery residents, but still about one-fourth of the trainees had never used a microscope, and one-fifth had never used an endoscope. Moreover, only about a quarter of trainees had used stereotaxic or intraoperative monitoring (Chaurasia et al., 2023; Srinivasan et al., 2022).

Recent advancements in robotic technology have generated the possibility of incorporating advanced technological tools to the neurosurgical operating room (Mattei et al., 2014). Nano techniques of (Abendroth et al., 2014) nanomanipulation, (Kanmounye et al., 2020) nanoimaging, (Kanmounye et al., 2022) nano scaffolds—non-surgical nano repair, and (Rukewe et al., 2017) nano neuromodulation, contribute to the recent advancement of neurosurgery (Andrews, 2009). In Low and Middle Income County (LMIC), when asked about research and conferences, 49.3% of neurosurgery residents were found to have no publication to their name, and 66.7% had never attended an international neurosurgery conference (Chaurasia et al., 2023). At the present time, neurosurgeons from many LMIC have produced no level 1 or level 2 evidence research publications. This represents a major limitation in the capabilities of our neurosurgeons. The deficiency in available literature is a consequence of insufficient government funding within the healthcare sector, while foreign residency programs offer enhanced research opportunities due to the availability of substantial research funding. Wide patient diversity in terms of ethnic, racial, cultural, language, geographical and religious is a benefit of foreign residency. Mentorship in this field is typically a long-term and multifaceted relationship, with mentors guiding their mentees from early in residency through the development of their independent careers. Overseas residency offers the opportunity to establish robust global networks with diverse neurosurgeons, notably since most international conferences are held in the USA. In addition, it cannot be denied training abroad provides way for in terms of finances and the modern standard of living attracts many international medical graduates. Additionally, 6.7% of students wished to train abroad because they felt foreign programs were more prestigious, while 3.4% wished to train abroad because their preferred specialty was not available for Post Graduate Medical Education (PGME) in their home country. In Greek and Botswana, some specialties do not have postgraduate training programs and in the other, there are more candidates for the available positions so students have long waiting periods before entry into preferred specialties (Rukewe et al., 2017). A review of Pakistan and its neighboring country's neurosurgical training program identified several deficiencies. No postgraduate trainees were interested in sub specialization in endoscopy, although an average of 17% of resident trainees indicated an interest in endoscopy. Lack of interest in sub specialization fields could be attributed to a lack of exposure to these fields and misinterpreting them as complicated or hard to pursue. The recruitment of foreign faculty as a part of College of Physicians and Surgeons Pakistan (CPSP) can significantly elevate training standards. (Srinivasan et al., 2022). The training programs in the developing countries need critical changes to provide favorable learning conditions with availability of appropriate surgical tools, structural changes of training programs, development of research interest, and improvement on the socioeconomic needs of the trainee (Chaurasia et al., 2023; Srinivasan et al., 2022; Chaurasia, 2023; Deora et al., 2020; Javed et al., 2023a, 2023b).

Final year medical students were approached in Cameroon, almost every participant (98%) expressed a firm desire to get into residency, 115 respondents (77.2%) intended to do their residency abroad, and 114 (76.5%) reported that they would come back home after their training abroad. Those who wished to return after their residency training stated the need to help the vulnerable (40.3%) and the need to serve their state (36.9%) (Kanmounye et al., 2020).

In summary, the choice to pursue neurosurgery training abroad may entail a temporary departure from one's homeland, but it is underpinned by a profound dedication to enhancing healthcare standards in many LMIC (Pahwa et al., 2023; Chaurasia, 2022). The knowledge and expertise acquired abroad are intended to benefit the people of LMIC by elevating the quality of neurosurgical care accessible to them. We firmly believe that through the acquisition of international education and experience, these individuals will be better positioned to make a substantial impact in the field of neurosurgery upon their return to homeland. Their pursuit of excellence is a testament to their commitment to improving our healthcare system.

## Conflict of interest

None.
